# Advances in understanding the cell types and approaches used for generating induced pluripotent stem cells

**DOI:** 10.1186/s13045-014-0050-z

**Published:** 2014-07-19

**Authors:** Jun Li, Wei Song, Guangjin Pan, Jun Zhou

**Affiliations:** 1 Department of Oncology, Shandong Provincial Hospital Affiliated to Shandong University, No. 324 Jingwu Weiqi Road, Jinan 250021, P.R. China; 2 Guangzhou Institutes of Biomedicine and Health, Chinese Academy of Science, 190 Kaiyuan Avenue, Science Park, Guangzhou 510530, P.R. China

**Keywords:** Induced pluripotent stem cells, Tissue origin, Approach, Generation

## Abstract

Successfully reprogramming somatic cells to a pluripotent state generates induced pluripotent stem (iPS) cells (or iPSCs), which have extensive self-renewal capacity like embryonic stem cells (ESCs). iPSCs can also generate daughter cells that can further undergo differentiation into various lineages or terminally differentiate to reach their final functional state. The discovery of how to produce iPSCs opened a new field of stem cell research with both intellectual and therapeutic benefits. The huge potential implications of disease-specific or patient-specific iPSCs have impelled scientists to solve problems hindering their applications in clinical medicine, especially the issues of convenience and safety. To determine the range of tissue types amenable to reprogramming as well as their particular characteristics, cells from three embryonic germ layers have been assessed, and the advantages that some tissue origins have over fibroblast origins concerning efficiency and accessibility have been elucidated. To provide safe iPSCs in an efficient and convenient way, the delivery systems and combinations of inducing factors as well as the chemicals used to generate iPSCs have also been significantly improved in addition to the efforts on finding better donor cells. Currently, iPSCs can be generated without c-Myc and Klf4 oncogenes, and non-viral delivery integration-free chemically mediated reprogramming methods have been successfully employed with relatively satisfactory efficiency. This paper will review recent advances in iPS technology by highlighting tissue origin and generation of iPSCs. The obstacles that need to be overcome for clinical applications of iPSCs are also discussed.

## At a glance

Induced pluripotent stem (iPS) cells (or iPSCs) have an extensive capacity for self-renewal, reproduction and differentiation, much like embryonic stem cells (ESCs).

Disease-specific or patient-specific iPSCs have both intellectual (e.g., disease modeling) and therapeutic benefits, and yet iPSCs face certain obstacles that hinder their applications in clinical medicine, especially the issues of convenience and safety.

Cells from three embryonic germ layers have been assessed to determine the range of tissue types amenable to reprogramming along with their particular characteristics. Some tissue origins have advantages over fibroblast origins with respect to efficiency and accessibility. Human urine-derived cells can be an attractive choice from which to generate iPSCs.

The delivery systems and combinations of inducing factors as well as the chemicals used to generate iPSCs have also been significantly improved to provide safe iPSCs in an efficient and convenient way. Small molecule strategy is much more promising because of its many advantages.

Extensive genetic screening and in vivo immunogenicity testing should be standard procedure to ensure the safety of human iPSCs prior to their clinical use.

## Introduction

Embryonic stem cells (ESCs), which are derived from the inner cell mass of mammalian blastocyst, have the ability to grow indefinitely while maintaining pluripotency and the ability to differentiate into cells of all three germ layers. Based on the hypothesis that factors that play important roles in the maintenance of ESC identity also play pivotal roles in the induction of pluripotency in somatic cells, in 2006, Takahashi et al. selected 24 candidate genes for inducing pluripotency in somatic cells under ESC culture conditions [[1]]. They successfully converted mouse embryonic fibroblasts (MEFs) and adult tail-tip fibroblasts (TTFs) to an undifferentiated state similar to that of ESCs through retroviral transduction of four transcription factors: Oct 3/4 (O), Sox2 (S), Klf4 (K), and c-Myc (M). These cells were termed induced pluripotent stem (iPS) cells (iPSCs). Subsequently, they generated human iPSCs (hiPSCs) from human dermal fibroblasts (HDFs) in the same way [[2]]. These hiPSCs were also generated by Yu et al. from fetal fibroblasts, newborn foreskin fibroblasts (BJ fibroblasts), and primary human fibroblast-like synoviocytes (HFLS) by introducing another four factors, namely, Oct3/4, Sox2, Nanog (N), and Lin28 (L), using lentiviral vectors [[3]].

Although the reprogramming process has been gradually revealed by intensive studies, the role of these transcription factors and the way they function in the acquisition and maintenance of pluripotency are still not fully understood. Simply speaking, the identity of somatic cells is strictly protected by an epigenetic barrier, and these cells acquire pluripotency when the reprogramming factors break the epigenetic barrier [[4]]. Possible mechanisms include silencing of retroviral transgenes upon establishment of pluripotency [[5]], reactivation of endogenous pluripotency genes [[1]], establishment of bivalent chromatin domains in the promoters of developmentally regulated genes [[6]], global DNA hypomethylation and DNA hypermethylation of imprinted gene loci [[7]], reactivation of the inactive X chromosome in female iPSCs and reorganization of chromatin fibers [[8]].

iPSCs are found to be similar to ES in their morphology, proliferation, surface antigens, gene expression, epigenetic status of their pluripotent cell-specific genes, and telomerase activity. Furthermore, these cells can differentiate into cell types of the three germ layers in vitro as well as into teratomas in vivo. They are also capable of germline transmission [[9]]. Nevertheless, iPSCs are superior to ESCs in some aspects because they have the potential to bypass both the practical and ethical concerns associated with ESCs. Unlike ESCs, which require the use of embryos and are thus complicated by ethical controversies and possible immune rejection, iPSCs can be derived from directed reprogramming of somatic cells from individual patients. Because the lifespan of fully differentiated cells is usually short and because such cells cannot self-renew (with a few exceptions, e.g., hepatocytes), iPS technology can provide them with an extensive ESC-like capacity to self-renew and the ability to generate daughter cells that can further differentiate into various lineages or terminally differentiate into a functional state, thus opening a new field of stem cell research. These disease-specific or patient-specific stem cells provide a unique opportunity to understand novel disease mechanisms [[10]], to screen for more effective and safe drugs [[11]], to develop autologous cell therapies including in vitro expansion and differentiation into cells of the hematopoietic lineage [[12],[13]], and to treat various diseases and injuries, such as Parkinson's disease [[14]] and cardiac damage [[15]].

Despite the potential implications of this technology, a number of questions about iPSC generation must be answered. There are several potential challenges to their possible clinical application in humans: (i) virus-mediated delivery of reprogramming factors permanently integrates transgenes into the human genome, potentially altering genomic features and risking viral transgene reactivation [[16]]; (ii) the reprogramming factors Klf4 and c-Myc are oncogenic and can potentially lead to the development of certain cancers [[17],[18]]; (iii) the low efficiency of iPS induction [[1]] and the lack of full and homogeneous iPS reprogramming processes are both problematic.

A series of major breakthroughs have been achieved. To determine the range of tissue types amenable to reprogramming, to decrease the tumorigenesis risk from transgene integration, and to increase the induction efficiency, investigators have extensively studied cells from three embryonic germ layers and identified some advantages of using tissues other than fibroblast. Delivery systems and combinations of inducing factors, as well as the chemicals used to generate iPSCs have also been improved; for example, iPSCs can now be generated without the use of the oncogenes c-Myc and Klf4 [[19]], and non-viral integration-free delivery methods are being successfully employed with reasonable efficiency. In this paper, we review recent advances in iPS technology, with a particular focus on tissue origin and generation of iPSCs.

### Tissue origins of iPSCs

Fibroblasts are the first cell type to have been successfully reprogrammed into iPSCs. Takahashi et al. tried 24 candidate genes and found that the retroviral transduction of OSKM reprogrammed MEFs and somatic TTFs into iPSCs [[1]]. These cells were also used to generate hiPSCs [[2]]. Fibroblasts are still widely used in iPSC technology, and their reprogramming efficiency and differentiation ability are the gold standard to which new methods must be compared.

Studying the strategy of iPSC production is different in different species due to practical and ethical concerns. iPSCs have been generated from mouse, rat [[20]], monkey [[21]], pig [[22]], dog [[23]], rabbit [[24]], and human cells. Pluripotency has been exhaustively analyzed in mice: iPSCs can form teratomas, which are differentiated tumors with tissues from all three embryonic germ layers. iPSCs contribute to all tissue types, including the germ line, when injected into murine blastocysts. iPSCs from MEFs could generate “all-iPSC mice” following injection into tetraploid blastocysts, thereby satisfying the most stringent criterion of pluripotency [[1]]. The same procedures cannot be applied to hiPSCs, which must rely on embryonic bodies and teratomas as the most stringent tests. This paper will only focus on iPSCs of mouse and human origin.

Cells from the three embryonic germ layers have been studied by researchers in the iPS field to determine the range of tissue types amenable to reprogramming, to decrease the tumorigenesis risk of transgene integration, and to increase the efficiency of induction. Malignant cell lines and primary cancer cells are also confirmed to be amenable to reprogramming by the iPS approach; however, this issue is not reviewed in this paper. To better understand the differences that result from the tissue origin of iPSCs, we will discuss them through two perspectives: differences within the same tissue origin and differences resulting from different tissue origins.

### Differences within the same tissue origin

#### Efficiency

The efficiency of iPSC induction is defined as the percentage of green fluorescent protein (GFP)-positive colonies with clear iPS morphology with a certain amount of mother cells transfected with transgenes. The efficiencies of iPSC induction from a single tissue of origin are different when using different technologies. It is hard to accurately correctly compare induction efficiency of different methods given differences in their experimental settings. Furthermore, efficiency is indeed an important criterion for an induction technology, but it is not always the most important one. Some technologies were improved to reduce integration of transgenes while sacrificing efficiency. In general, there are a few lessons regarding the efficiencies of iPSC induction from a single tissue origin: (i) efficiency is higher in mice than in human, successful induction using some technologies has not been reported in humans due to very low efficiency; (ii) decreasing the number of transgenes with the same methods would reduce the induction efficiency by orders of magnitude; (iii) non-integration methods are much less efficient than integration methods, with the exception of the Sendai virus (SeV) versus retrovirus and lentivirus vectors; (iv) certain chemicals and microRNAs (miRNAs) can also markedly improve the efficiency. A detailed summary of the efficiencies of different induction methods using fetal and somatic fibroblasts are listed chronologically in Table 1 according to the publication date of descriptive papers, which straightforwardly represents the development of the technology.

**Table 1:** Efficiency of different iPS induction methods in fibroblasts

**Delivery system**	**Species**	**Technology**	**Efficiency (%)**	**reference**
Virus	M	OSKM/retrovirus	0.001 ~ 0.01	[[1]]
	H	OSKM/retrovirus	0.02	[[2]]
	H	OSNL/lentivirus	0.01 ~ 0.02	[[3]]
	M, H	OSK/retrovirus	<0.001	[[25]]
	M	OSK/retrovirus + VPA	2	[[26]]
	H	OSK/retrovirus + VPA	1	[[19]]
		OS/retrovirus + VPA	<0.001	
	M	OSKM/adenovirus	0.0001 ~ 0.001	[[27]]
	H	OSKM/retrovirus/p53 siRNA and UTF1	2	[[28]]
	M	OSKM/a single polycistronic retrovirus	0.0001	[[29]]
	M	OSKM/retrovirus/p53 siRNA	20	[[30]]
		OSK/retrovirus/p53 siRNA	10	
	H	OSKM/SeV	~1%	[[31]]
	M	pMX-OSKM cDNA/retrovirus + Vc	8.75	[[32]]
	H	OSKM/retrovirus + VPA + Vc	6.2	[[33]]
Plasmids	M	pCX-cMyc + pCX-OSK-2A	0.0001 ~ 0.0003	[[34]]
EV	H	IRES2 -mediated OSNLKM + SV40LT	0.0003-0.0006	[[35]]
Protein	M	OSKM + VPA/recombinant protein	0.006	[[36]]
		OSK + VPA/recombinant protein	0.001	
	H	OSKM/cell extract	0.001	[[37]]
Modified-RNA	H	OSKML	2(KSOML), 1.4(KSOM)	[[38]]
Minicircle DNA	H	OSNL	~0.0005	[[39]]
Artificial chromosome vectors	H	OSKM	0.001%.	[[40]]
Small-molecule compounds	M	7 small-molecule compounds	0.2	[[41]]

Abbreviations: *M* mouse, *H* human, *oriP/EBNA1* Epstein-Barr nuclear antigen-1, *EV* episomal vectors, *IRES2* internal ribosome entry site 2, *SV40LT* SV40 large T gene, *VPA* valproic acid, *Vc* vitamin C, *siRNA* small-interfering RNA.

Another example of different efficiencies in the same tissue origin in response to different induction technologies is found in human adipose stem cells (hASCs). Sun et al. [[42]] reported that when hASCs are transduced with individual lentiviruses containing OSKM, the incidence of ESC-like colonies is 0.2%, whereas Vc or Vc + VPA with retroviral pMX vectors containing OSKM cDNAs reprogrammed hASCs with a much higher efficiency (up to 7.06%) [[32]], nevertheless, the reprogramming efficiency is much lower with minicircle DNA, which contains a single cassette of OSNL plus a GFP reporter gene separated by self-cleavage peptide 2A sequences. This system yields an overall reprogramming efficiency of ~0.005% [[39]].

Some small molecules can increase the efficiency of reprogramming primary human fibroblasts to a pluripotent state [[26]]. When the same three-factor combination

(OSK) via retroviral transduction is used, the addition of VPA improves reprogramming efficiency by a factor of 1,000-fold. Furthermore, VPA could enable reprogramming with only two factors (Oct4 and Sox2) with efficiency similar to that of three factors, suggesting that VPA treatment effectively dispenses the need for Klf4. Other small molecules that could obviate the need for certain exogenous factors will be reviewed below.

#### Somatic coding mutations

Somatic coding mutations of iPSCs are different even with the same cell origin. The majority of protein-coding exons (exomes) in the 22 hiPS cell lines reprogrammed using five different methods were sequenced. Three of these lines had been produced via integrating methods (four-factor retroviral, four-factor lentiviral and three-factor retroviral) and two non-integrating methods (EV and messenger RNA (mRNA) delivery into the fibroblasts) in seven laboratories and from nine matched fibroblast lines [[43]]. It was found that these cell lines contained an average of five protein-coding point mutations in the regions sampled (with an estimated six protein-coding point mutations per exome). The majorities of these mutations are non-synonymous, nonsense, or splice variants and are enriched in genes that have been associated with cancers. At least half of these reprogramming-associated mutations are found to pre-exist in fibroblast progenitors at low frequency, while the rest occur during or after reprogramming. It should be considered whether some of these mutations could increase the risk of disease when hiPS-cell-derived cells/tissues are used in the clinic. Although the functional effects of the mutations remain to be characterized experimentally, it is nonetheless striking that the observed reprogramming-associated mutational load shares many similarities with characteristics observed in cancer. Furthermore, the observation of mutated genes involved in human Mendelian disorders suggests that the risk of diseases other than cancer should be evaluated as well for hiPS-cell-based therapeutic methods. Thus, although all hiPSC lines are extensively characterized for pluripotency and have normal karyotypes before DNA extraction, pre-existing and new mutations occur during and after reprogramming. These mutations can produce genetic and epigenetic changes in the hiPSCs such that extensive genetic screening should become a standard procedure to ensure the safety of hiPSCs before clinical use. One corollary is that if reprogramming efficiency is improved to a level such that no colony picking and clonal expansion is necessary, the resultant hiPSCs could potentially be free of mutations.

#### Copy number variations (CNVs)

Significantly more CNVs are present in early-passage hiPSCs than in intermediate passage hiPSCs established either by retroviral or piggyBac (PB) transposon delivery methods [[44]]. Fortunately, most CNVs render the affected cells at a selective disadvantage; thus remarkably, the expansion of hiPSCs in culture selects rapidly against mutated cells, driving the lines toward a genetic state resembling human ESCs.

### Differences resulting from different tissue origins

#### Accessibility and universality

There is still no consensus regarding the preferred tissue from which to harvest donor cells for iPSC reprogramming. The ideal cell source should be easily accessible, susceptible, and universal (any age, sex, ethnic group, and body condition). Different cell sources are associated with distinct advantages and disadvantages.

Dermal fibroblasts are currently the most frequently used cells for reprogramming, but their obtainment requires biopsy, and candidates sometimes refuse to donate the necessary tissue. Additionally, the procedure is contraindicated in life-threatening skin diseases (e.g., severe epidermolysis bullosa) or burn cases. Keratinocytes have similar limitations [[45]]. They are keratin-dense epithelial cells and generate the outer protective epidermal barrier of the skin surface as well as appendages such as hair and nails. hASCs [[42]], a heterogeneous group of multipotent progenitor cells similar to the ones from fibroblast and the keratinocytic lineage can be readily and safely derived from adipose tissue of adult humans in very large quantities by lipoaspiration without extended time for expansion, but lipoaspiration is invasive and sometimes harmful.

It is unclear if dermal papilla (DP) cells [[46]], which are specialized skin fibroblasts that are considered to instruct hair follicle stem cells, are a good source for reprogramming because the growth and quality of hair follicles depend on age, genotype, and medical conditions of the human donors, although they are reprogrammed more efficiently than skin and embryonic fibroblasts due to high endogenous expression of Sox2 and c-Myc.

Blood cells are among the most favorable cell types for iPSC induction, but their development has thus far been disappointing. They include stem/progenitor cells and terminal blood cells. Hematopoietic stem/progenitor cells are young cells that are expected to carry minimal somatic mutations and possess the immunological immaturity of newborn cells. They are rare in peripheral blood but are naturally rich in the bone marrow (BM), umbilical cord blood (CB), and placenta. Harvesting mobilized CD34+ cells isolated from peripheral blood is a cumbersome, expensive, and time-consuming process [[47]] and granulocyte colony stimulating factor (G-CSF) mobilization is a procedure that is frequently associated with significant side effects, including bone pain, headache, fatigue, and nausea [[48]]. BM harvesting is an invasive procedure [[49]]. Other choices are the CB and the placenta [[50]], which are extra-embryonic tissues of particular interest in regenerative medicine. They share an early developmental origin and are a source of vast amounts of cells with multilineage differentiation potential. They are also poorly immunogenic and, unlike many other stem-cell sources, are not controversial. Moreover, these cells are likely exempt from incorporated mutations when compared with juvenile or adult donor cells such as skin fibroblasts or keratinocytes. Unfortunately, they are only available for a minority of individuals who have had their samples banked at birth.

Peripheral blood cells (PBCs) can be isolated by routine venipuncture with minimal risk to the donor and can be obtained in sufficient numbers to enable reprogramming without the need for prolonged expansion in culture. More importantly, PBCs provide convenient access to numerous patient samples stored in blood banks. Frozen blood samples, when reprogrammed to iPSCs, are of major interest because they would allow for retrospective molecular analysis of rare diseases. However, some disadvantages associated with the blood specimens restrict their usage for generation of iPSCs. First, the efficiency of this cell source is extremely low (0.0008 to 0.001%). Second, the main parent cells are mature T cells bearing specific T cell receptor (TCR) re-arrangements [[51],[52]], which yield iPSCs with germ line IgH and TCR alleles, that are undesirable for some potential applications in regenerative medicine. Moreover, in cases where blood infections are involved (e.g., hepatitis C virus, and HIV), giving blood is not exempt of concerns. Finally, reprogramming may be problematic in patients with blood diseases (e.g., hemophilia and leukemia).

Human urine -derived cells (HUCs) are another favorable iPSC candidate [[53],[54]], in particular because harvesting is completely non-invasive. In normal physiology, approximately 2000 to 7000 cells from an extensive network of tubules in the kidney and downstream parts of the urinary tract (the ureters, bladder, and urethra) become detached into the urine daily. They can be collected anywhere without medical assistance and are easily expanded. Their efficiency with retroviral pMX vectors containing the cDNAs of human OSKM is between 0.1 and 4%, while their efficiency with oriP/EBNA EVs carrying a combination of reprogramming factors encoded by OSK, SV40LT, and the miRNA cluster MIR302–367 through electroporation is approximately 0.2%. These are quite high efficiencies for the integration-free reprogramming method. Therefore, urine samples may be considered a preferred source for iPSC derivation. However, other reports indicate that the number of excreted cells range from 0 to 6500 showing a high degree of individual variation, and cell proliferation is achieved in only approximately 1/3 of the samples [[55]]. This raises the questions whether HUCs can be universally applied for generating iPSCs. Moreover, culturing HUCs usually requires long periods of time (3 ~ 6 days or more to form the HUC clones, 7 ~ 10 days or more for the clones to spread over the dish) prior to the introduction of reprogramming factors into passage 2 ~ 4 HUCs [[53]]. Furthermore, although HUCs may produce iPSCs bearing fewer somatic cell mutations and copy number variations than iPSCs from the skin due to reduced direct exposure to radiation, iPSCs derived from this source have not yet been fully analyzed for the spectrum of mutations and other characteristics of iPSCs.

Cells of some tissue origins are excluded from easily-accessible cell resources because they are difficult to reach, with potential risks due to their positions in the body. These cell types include neural stem cells (NSCs), melanocytes, meningiocytes, hepatocytes, gastric epithelial cells, and pancreatic β cells.

#### Efficiency

Parent cells of different tissue origins have different efficiencies. Researchers have tried to generate iPSCs from derivatives of the three embryonic germ layers and have found that the parental cells of different tissues of origin have different efficiencies. Some cell types are difficult to reprogram even with a virus-mediated method, let alone a non-integrating method. It is conceivable that epithelial cells are more amenable to reprogramming, perhaps because unlike fibroblasts, they need not undergo a mesenchymal-to-epithelial transition to produce iPSCs. Nevertheless, the high endogenous levels of transgenes required for iPSC generation in parent cells may also be associated with transcriptional and epigenetic states that are favorable to reprogramming. A detailed summary of the efficiency of various cell origins other than fibroblasts is shown in Table 2.

**Table 2:** The efficiency of cells of various non-fibroblast origins for generating iPSCs

**Germ layer**	**Cells**	**Methods**	**Efficiency (%)**	**Reference**
Mesoderm	Human fibroblast-like synoviocytes	OSKM/retrovirus	0.002	[[2]]
	hASCs	OSKM/lentivirus	0.4	[[42]]
	hASCs	OSKM + VPA + Vc/retrovirus	7.06	[[32]]
	hASCs	OSNL cassette/minicircle DNA	~0.005	[[39]]
	Immature B lymphocytes of mouse iPS chimeras	Carry dox-inducible OSKM retroviruses already	-	[[56]]
	Mature B lymphocytes of mouse in the iPS chimeras	Carry dox-inducible OSKM retroviruses already + C/EBPalpha) or Pax 5 knockdown	1/30	
	Terminally differentiated T lymphocytes of p53-null mouse	OSKM/retrovirus	0.00015	[[30]]
	G-CSF mobilized human CD34+ PBCs	OSKM/retrovirus	0.01 ~ 0.02	[[47]]
			0.002	
	Human CB-derived ECs	OSNL/lentivirus	0.01 ~ 0.03	[[50]]
	Mouse BW progenitor cells	OSKM/retrovirus	0.00002 ~ 0.00006	[[49]]
	Human CD133+ CB cells	OSKM,OSK,OS/retrovirus	0.002 ~ 0.007(OSK)	[[57]]
	Human PBMCs (T cell and myeloid cell)	OSKM polycistronic expression cassette/dox-inducible lentivirus	0.001 ~ 0.0002	[[51]]
	Human PB CD34+ cells	OSKM/two rounds of lentiviral infection	0.002	[[52]]
	Human T cell in PBMCs		0.0008 ~ 0.001	
	Human and murine CMCs and periosteal membrane	pMX-OSKM cDNA/retrovirus + Vc + VPA	higher than UMCs	[[32]]
	Human UMCs	pMX-OSKM cDNA/retrovirus + Vc + VPA	0.4	[[33]]
	Human AMCs		0.1	
	Oral mucosa fibroblasts	OSKM/retrovirus	0.022	[[58]]
Endoderm	Mouse liver and stomach cells	OSKM/retrovirus	_	[[59]]
	Mouse pancreatic β cells	OSKM/inducible lentivirus	0.1 ~ 0.2	[[60]]
	HUCs	Retroviral pMX vectors containing the cDNAs of mouse OSKM	0.1 ~ 4	[[53]]
	HUCs	OSK + SV40LT + MIR302–367/EV electroporation	0.2	[[54]]
	Human nasal epithelial cells	OSKM/SeV	0.08 ~ 0.10	[[61]]
	Human dental pulp cells	OSK/retrovirus	0.01 ~ 0.10	[[62]]
Ectoderm	Mouse NSCs	OSKM/retrovirus	3.6	[[63]]
		Oct4 with either Klf4 or c-Myc/retrovirus	0.11	
		Oct4/retrovirus	0.014	[[64]]
	Mouse meningiocytes	pMX of OSKM-cDNAs/retroviral	0.8	[[65]]
	Human keratinocytes	OSKM/retrovirus	1	[[45]]
		OSKM/a single polycistronic retrovirus	-	[[29]]
	Mouse and human melanocytes	OKM or OKS/dox-inducible lentivirus	0.19	[[66]]
	Human DP cells from hair follicles	OSKM/pMX-based retrovirus	1.38	[[46]]
		OK/pMX-based retrovirus	0.024	
Chimeras	Cells of mouse iPS-cell chimeras	OSKM/dox-inducible polycistronic lentiviruses	_	[[67]]

dox: doxycycline; CMCs: chorionic mesenchymal cells; UMCs: mesenchymal-like cells from the umbilical cord matrix; AMCs: the amniotic membrane mesenchyme.

#### Number of exogenous factors

Parent cells of different tissue origins require a different number of exogenous factors. Considering that the ectopic expression of c-Myc causes tumorigenicity in offspring and that retroviruses themselves can cause insertional mutagenesis, iPSCs generated with a minimal number of factors were studied to identify cell types that can be more easily reprogrammed with fewer factors and with higher efficiency to increase the likelihood of ultimately replacing the remaining factors with small molecules.

Mouse and human fibroblasts can be reprogrammed into iPSCs in the absence of the c-Myc retrovirus, although their efficiency is as low as <0.001% [[25]]. Furthermore, it is believed that c-Myc plays a much smaller role in the generation of iPS-Hep and iPS-Stm cells than in iPS-fibroblasts [[59]]. This is because the omission of c-Myc decreased the colony numbers to 20 to 40% of those obtained by the four factors, in contrast to the generation of iPS-fibroblasts, which caused a 90% decrease in colony numbers in response to c-Myc omission.

Adult mouse NSCs express higher endogenous levels of Sox2 and c-Myc than ESCs. Exogenous Oct4 in combination with either Klf4 or c-Myc is found to be sufficient to generate iPSCs from NSCs [[63]], while Oct4 alone is confirmed to be both necessary and sufficient [[64]]. However, although this approach is potentially applicable for generating hiPSCs, it is limited by the difficulty and potential risks of obtaining NSCs from the brains of patients.

Melanocytes are, like NSCs, of neuroectodermal origin and hence might require fewer factors for their conversion into iPSCs. Interestingly, with dox-inducible lentiviral vectors expressing OSKM, the efficiency of reprogramming melanocytes is more than 3-fold higher than that of fibroblasts (0.19% versus 0.056%); this efficiency decreases to 0.03% with all three factors (with OKM) and to 0.02% in the absence of ectopic c-Myc, which is approximately 10-fold lower than the efficiency of melanocyte reprogramming in the presence of c-Myc and fibroblast reprogramming [[63]].

#### Epigenetic memory

iPSCs of different tissue origins are discovered to have different epigenetic memories when analyzed for DNA methylation. iPSCs derived from non-hematopoietic cells (neural progenitors and fibroblasts) are found to retain residual methylation at various loci where demethylation is required for a hematopoietic fate, as a result, they manifest a reduced blood-forming potential in vitro [[68],[69]]. Epigenetic memory has a dual nature. For some applications, the epigenetic memory of the donor cell may be advantageous when the directed differentiation to specific tissue fates is challenging; for others, epigenetic memory can restrict the use of the resultant iPSCs in regenerative medicine. For example, iPSCs generated from T cells from human PBMCs still contain TCR, restricting their broad application in regenerative medicine [[52]].

#### Differentiation potential

The tissue origin of iPSCs affects their differentiation potential [[70]]. For example, the teratoma-forming propensity is different of secondary neurospheres generated from 36 mouse iPSC lines derived in 11 different ways. These iPSC lines can be characterized by (i) their origin (e.g., MEF, TTF, hepatocyte, or gastric epithelial cell); (ii) the presence or absence of c-Myc retroviral transduction; (iii) the presence or absence of drug selection for Nanog or Fbxo15 expression. Retrovirus-derived iPSCs from adult TTFs and hepatocytes could contribute to chimeric mice; however, when the iPSCs are induced in vitro to differentiate into neural cells, they frequently form teratomas following cell transplantation. In contrast, iPSCs prepared from MEFs show low teratoma formation, similar to ESCs.

Furthermore, the differentiation propensity and methylation profile of iPSCs can be reset. When blood-deficient neural progenitor-derived iPSCs are differentiated into blood cells, which are then reprogrammed into iPSCs, their blood-forming potential is markedly increased [[69]].

#### Susceptibility to tumorigenesis

The use of viruses encoding the reprogramming factors contributes undeniably to the tumorigenesis of iPSCs because even low vector expression may alter the differentiation potential of iPSCs or induce malignant transformation, and viral transgenes could also be integrated into the somatic genome. For example, mouse iPSCs generated from retrovirus-mediated reprogramming of fibroblasts give rise to adult chimeras and show competence for germline transmission; however, the chimeras and progeny mice derived from iPSCs frequently develop tumors [[16]]. However, tissue sources of iPSCs seem to play a role in their susceptibility to tumorigenesis through the viral-integrating method. Just as in mice and via the same methods, hepatocytes and gastric epithelial cells [[59]] appear not only to be more easily reprogrammed but also require fewer retroviral integrations than fibroblasts. No increased tumorigenicity was observed in chimera mice derived from iPS-Hep and iPS-Stm cells evaluated up to 30 weeks.

Somatic coding mutations of iPSCs have been confirmed to be different even with the same cell origin of different fibroblast lines when different integrating and non-integrating methods are used [[43]]. However, no data exists regarding somatic coding mutations of iPSCs of different cell origin when the same non-viral, integration-free delivery methods are used.

### Improvements in the approaches for iPSC generation

The currently available methods for iPSC induction can be divided into four categories based on their vector types: viruses, DNA and RNA (plasmid, episomal plasmid, and transposon), cell penetrating peptides, and chemicals. Although retroviral and lentiviral vectors have a high reprogramming efficiency, the use of viruses encoding reprogramming factors represents a major limitation of the technology because even low vector expression may alter the differentiation potential of iPSCs or induce malignant transformation, while viral transgenes can also be integrated into the somatic genome. Several groups have made considerable progress to overcome such technical problems. iPSCs can be generated without the oncogenes c-Myc and Klf4 [[26]]. Furthermore, genome-integrating but excisable systems have worked to remove the integrated transgenes [[71],[72]]. Transient expression of reprogramming factors with adenoviral [[27]], SeV [[31]], and non-viral delivery integration-free methods such as plasmid [[34]], EV [[35]], proteins [[36],[37]], and chemicals [[41]] have been successfully employed, albeit at a very low efficiency (with the exception of SeV). Nevertheless, further study should focus on whether there is any difference in the characteristics of iPSCs established by different methods prior to their eventual use in medical applications.

### Viral induction of iPSCs

According to the original method of iPS generation, cells were reprogrammed into an ESC-like state by viral transduction with defined combinations of factors. Thomson et al. [[9]] found that each iPS clone contained three to six retroviral integrations for each factor. Thus, each clone had more than 20 retroviral integration sites in total, which may increase the risk of tumorigenesis. In contrast, c-Myc is known as a proto-oncogene, and its reactivation may give rise to transgene-derived tumor formation [[16]]. Furthermore, although reprogramming has been achieved in the absence of c-Myc [[25]], the remaining integrated reprogramming factors could also cause tumor formation [[73]]. To apply this technology to cell transplantation therapy, it is crucial to generate iPSCs with fewer viruses and transgenes.

#### Induction with varying numbers of transgenes

Transgene numbers can be decreased by changing the parent cell type. Considering that the ectopic expression of transgenes can cause tumorigenicity in offspring, the generation of iPSCs with a minimal number of factors has been extensively studied. Indeed, studies have shown that it is possible to generate iPSCs in the absence of c-Myc with retroviruses from mouse and human fibroblasts [[25]] and from mouse hepatocyte and stomach cells [[59]], to generate pluripotent stem cells with only oct4 from adult mouse NSCs [[64]], and to generate pluripotency with dox-inducible lentiviral vectors expressing OSK or OKM from melanocytes [[66]].

Some small molecules could also replace certain exogenous factors. When reprogramming primary human fibroblasts to a pluripotent state, VPA enables reprogramming with OS alone with an efficiency similar to that of OSK, suggesting that VPA treatment effectively obviates the need for Klf4 [[23]]. Kenpaullone could activate Nanog expression in mouse fibroblasts and substitute for Klf4, although the underlying mechanism remains unclear [[74]]. A specific glycogen synthase kinase 3 (GSK-3) inhibitor, CHIR99021, can induce reprogramming of MEFs transduced with only OK. When combined with Parnate (also known as tranylcypromine), an inhibitor of lysine-specific demethylase 1, namely CHIR99021, human primary keratinocytes can also be reprogrammed through transduction with OK [[75]]. The transient expression of OCT4 alone with the use of VPA and BIX01294, a histone methyltransferase G9a inhibitor, is sufficient to allow for reprogramming of NSCs [[76]]. In addition, the pan-Src family kinase (SFK) inhibitors Dasatinib and PP1 are able to replace Sox2 for the generation of iPSCs from MEF [[77]].

Hou et al. identified a specific chemical combination of four small molecules: VPA, tranylcypromine, CHIR99021, and 616452 (VC6T). These four molecules are sufficient to permit the reprogramming of mouse embryonic and adult fibroblasts in the presence of a single transcription factor, Oct4, which can replace SKM. Two years later, they generated iPSCs using only seven chemicals [[41]].

### Drug-inducible transgenic systems

Induced pluripotency is complicated by the need for infection with high-titer retroviral vectors, which produce genetically heterogeneous cell populations. Wernig et al. generated iPSC chimeras and produced genetically homogeneous ‘secondary’ somatic cells that carry reprogramming factors defined as dox-inducible transgenes [[67]]. A drug-inducible transgenic system could directly reprogram multiple ‘secondary’ somatic cell types without the need for a virus. These cells could be reprogrammed upon dox exposure without the need for viral infection with 25- to 50-fold greater efficiencies than those observed using direct infection and drug selection for pluripotency marker reactivation.

Technically speaking, this method should not be categorized as a non-virus induction method because primary fibroblasts are infected with dox-inducible lentiviruses encoding OSKM and chimeric mice are generated after blastocyst injection of iPSCs. Nevertheless, this drug-inducible transgenic system is quite useful in transfecting cells and convenient for screening out cells that are not transfected by transgenes.

### Single polycistronic vector

The nucleofection of a single polycistronic vector to generate iPSCs can reduce transgene insertion into the genome. A major impediment to the use of iPSCs for therapeutic purposes has been the viral-based delivery of reprogramming factors; multiple proviral integrations pose a greater danger of insertional mutagenesis. On the other hand, the cellular and genetic heterogeneity of fibroblasts randomly infected with a single transgene coded by a separate virus complicates the exploration of important molecular events occurring during reprogramming and limits the scalability required for high-throughput analyses. Carey et al. reported an approach to deliver up to four reprogramming factors in a single virus using 2A “self-cleaving” peptides. These peptides support efficient polycistronic expression from a single promoter with a single proviral copy in both embryonic and adult somatic mouse cells as well as in human keratinocytes [[29]]. These iPSC lines show no evidence of transgene insertion into their genomes. Most of all, the single polycistronic vector could generate genetically identical cell populations amenable to reprogramming without any further genetic interference. Several similar methods [[78]–[80]] have been reported in the same year, and these methods are widely used to this day.

### An integrating but excisable system

To derive hiPSCs that were free of proviruses, Soldner et al. used dox-inducible lentiviral vectors that could be excised after integration with Cre-recombinase [[71]]. Vectors transducing either OSKM or OSK are used to generate hiPSCs after being transiently cotransfected with plasmids for Cre-recombinase and EGFP and subsequently EGFP-positive and Cre-expressing cells are sorted. The excision of all reprogramming factors is confirmed by Southern blot analysis with a different restriction digestion. Furthermore, polymerase chain reaction (PCR) of the genomic DNA with primers specific for Cre-recombinase confirms that none of the clones has stably integrated the electroporated plasmids. However, it should be noted that a number of technical hurdles complicate the use of Cre-mediated DNA excision from stem cells, potentially limiting the application of these methods to easily generate iPSCs free of floxed transgenes. First, the delivery of Cre to ESCs or iPSCs is known to be inefficient. Second, screening methods to detect successful Cre-recombination may be cumbersome. Finally, the clumping of cells after the delivery of Cre may result in mosaic colonies containing some cells that have failed to undergo Cre-recombination.

Sommer et al. improved the method of Soldner et al. [[72]]. A novel version of their single polycistronic vector containing a reporter fluorochrome allows for the direct visualization of vector excision in living iPSCs in real time. Cre-recombination efficiency and the excision of reprogramming transgenes from the resulting iPSCs could be readily visualized and monitored in individual living cells and colonies in culture. In addition, through the excision of only a single vector copy, their approach minimizes the risk of chromosomal translocations, which is a significant advance over prior methods. Furthermore, a direct comparison of iPSC clones before and after excision reveals that the removal of the reprogramming vector markedly improves the developmental potential and differentiation capacity of the iPSCs. It should be emphasized that their approach leaves approximately 200 base pairs (bp) of exogenous DNA, primarily viral long terminal repeat (LTR), behind in the iPSC genome. Although the viral LTR is inactivated in their studies and possesses no intrinsic promoter/enhancer activity, there is still a theoretical risk that insertional mutagenesis may arise from this genomically integrated exogenous DNA.

Papapetrou et al. further improved the above methods [[81]] and developed a strategy that includes additional steps for the mapping of the integration sites in the genome. Their method also allows for the selection of clones with few or a single appropriate site for the integration of the residual LTR.

### Adenoviral vector

Adenoviral vectors allow for transient, high-level expression of exogenous genes without the integration of such genes into the host genome. Stadtfeld et al. generated mouse iPSCs (adeno-iPSCs) from fibroblasts and liver cells [[27]] at extremely low efficiency of 0.0001% to 0.001%, which is significantly lower than that obtained with integrating viruses (~0.01 to 0.1%). This is most likely because many cells do not maintain viral expression long enough to trigger entry into a state sustained by endogenous pluripotency factors.

PCR analysis of genomic DNA isolated from adeno-iPSC clones excludes the possibility of permanent viral integration. Southern blot analysis using the cDNAs of the four viral vectors carrying OSKM as probes confirmed the PCR results and yielded no evidence for the continuous presence of adenoviral sequences in the adeno-iPSCs. Although highly unlikely, they cannot rule out the possibility that small pieces of adenoviral DNA have inserted into the genome of adeno-iPSCs but were not observed given the detection limits of Southern blot analysis.

A difference in these adeno-iPSCs is that DNA content analysis showed that 3 out of 13 (or approximately 23%) adeno-iPSC lines were tetraploid, which is not observed in iPS cells produced with retroviral or lentiviral vectors.

### SeV vector

SeV, a RNA virus carrying no risk of altering the host genome, is shown to be an efficient solution for generating safe iPSCs in the work of Fusaki et al. [[31]]. SeV-derived transgenes are decreased during cell division; moreover, viruses could be easily removed by antibody-mediated negative selection utilizing the cell surface marker HN that is expressed on SeV-infected cells. SeV is shown to be an ideal vector for generating hiPSC. First, as emphasized repeatedly, the SeV vector allows the expression of transgenes without the risk of modifying the host genome. Second, the efficiency of iPSC generation by gene transduction with SeV vectors is significantly higher (~1%) compared with other methods, especially without any transfection reagents or chemicals. Finally, it is easy to select iPSCs that deplete the viral genome from the cytoplasm.

Although the use of SeV requires stringent steps to purge the reprogrammed cells of replicating virus and the sensitivity of the viral RNA replicase to transgene sequence content may limit the generality of this reprogramming vehicle, this method is still used widely and can be used to efficiently deliver up to four exogenous genes into various mammalian cells, including primary tissue cells and human hematopoietic stem cells [[82]–[86]].

### Non-viral induction of iPSCs

The development of novel approaches for generating integration-free iPSCs has eliminated concerns about integrating virus-associated genotoxicity in clinical applications. Among the virus-free method, PB transposons require a second step to remove the transgenes once reprogramming has been achieved. Other one-step non-virus approaches make use of plasmids [[34]], minicircle DNAs [[39]], human artificial chromosome vectors (HACs) [[40]], protein transduction [[36],[37]], and chemicals [[41]], but are very inefficient in generating integration-free iPSCs. In contrast, the use of modified mRNA [[38]] and oriP/EBNA1-based EV [[35]] represent relatively efficient approaches that have been readily reproduced in different laboratories. The current most cost-effective approach is EV because it does not require the packaging of viral vectors and only needs simple infection instead of daily or multiple additions of factors for successful reprogramming. However, small molecule strategy is much more promising because of its many advantages.

### PB transposons

DNA transposons are genetic elements that can relocate between genomic sites via a “cut and paste” mechanism. PB is originally isolated from Trichoplusia ni and subsequently found to be a host-factor-independent method for efficient transposition in many different species. The PB transposon/transposase system requires only the inverted terminal repeats flanking a transgene and transient expression of the transposase to catalyze insertion or excision events. One important feature of the PB transposon is that it nearly always excises itself precisely, leaving no footprint behind [[87]].

Woltjen et al. demonstrated the successful and efficient reprogramming of murine and human embryonic fibroblasts using the dox-inducible OSKM transcription factor delivered by PB transposition [[88]]. By taking advantage of the natural propensity of the PB system for seamless excision, the individual insertions can be removed without a trace from the established iPSC lines, providing an invaluable tool for discovery. The successful transposon-based reprogramming of fibroblasts to iPSCs represents a significant advance in the methods of transgene delivery. First, PB transposition permits technical simplification and improved accessibility of reprogramming methodology, making use of effortless plasmid DNA preparation and commercial transfection products for delivery. This eliminates the need for specialized biohazard containment facilities and the production of high-titer, limited-lifetime viral stocks. Second, the range of somatic cell types that could be used for reprogramming is not limited by a decreased susceptibility to viral infection. Third, PB-mediated delivery will allow the option of xeno-free hiPSC production, contrary to current viral production protocols that use xenobiotic conditions. Finally, accurate transgene removal through transposase expression has been demonstrated in various cell lines. However, it is disadvantaged by labor intensive removal of multiple transposons.

Meanwhile, in the same journal, Kaji et al. reported that the non-viral transfection of a single multiprotein expression vector, the 2A-peptide-linked reprogramming cassette MKOS-ires-mOrange, could reprogram both mouse and human fibroblasts [[89]]. Moreover, the transgene could be removed once reprogramming had been achieved, when the single vector reprogramming system is combined with the PB transposon.

Although the PB transposon is thought to nearly always excise itself precisely, leaving behind no footprint, it is still found that significantly more CNVs are present in early-passage than in intermediate passage hiPSCs, as has been established by retroviral delivery methods [[44]]. Fortunately, most CNVs are a disadvantage to the affected cells. Remarkably, the expansion of hiPSCs in culture rapidly selects against mutated cells, driving the lines toward a genetic state resembling that of human ES cells.

### Plasmid vector

Okita et al. placed cDNAs encoding OSK into a single expression vector and c-Myc into another plasmid (pCX-cMyc) and observed the highest efficiency of GFP-positive colony formation when the factors were in the order of OKS [[34]]. Interestingly, when they transfect pCX-OKS-2A on days 1 and 3, and pCX-cMyc on days 2 and 4, PCR analyses detected plasmid incorporation into the host genome. However, after the transfection protocol was modified to transfect pCX-OKS-2A and pCX-cMyc together on days 1, 3, 5, and 7, southern blot analyses did not detect any integration of the transgenes into the clones, while two of 11 GFP-positive clones were observed with amplification of the plasmid DNA. Nevertheless, the efficiency of the establishment of plasmid-iPSCs is very low; in 2010, the same group again reported that the efficiency is 0.0001 ~ 0.0003%, and this method is only applied to MEFs [[90]].

### OriP/EBNA1 EV

Yu et al. reported that hiPSCs completely free of vector and transgene sequences could be derived from fibroblasts by a single transfection with oriP/EBNA1-based EV [[35]]. Derived from the Epstein-Barr virus, oriP/EBNA1 vectors are well suited for introducing reprogramming factors into human somatic cells. These plasmids can be transfected without the need for viral packaging and can be subsequently removed from cells by culturing in the absence of drug selection. One problem is that the stable transfection efficiency of the oriP/EBNA1-based vector is almost two orders of magnitude less than that of the lentiviral vector OSNL in human newborn fibroblasts (HNFs). To overcome this problem, they used internal ribosome entry site 2 (IRES2)-mediated expression of OSNL with oriP/EBNA1-based vectors, and the reprogramming efficiency is improved by approximately 10-fold (~0.1%). The addition of c-Myc and Klf4 further improves the reprogramming efficiency to over 1%. When they cloned all six reprogramming factors (OSNLKM) into an oriP/EBNA1 vector using IRES2 for coexpression, they initially failed to yield hiPSC colonies; possibly due to the toxic effects of high level c-Myc expression. They next included the SV40 large T gene (SV40LT) and produced iPSCs with a low efficiency (0.0003 ~ 0.0006).

With EV, exogenous DNA is not integrated into the hiPSC genome. Due to the gradual loss of cellular EV in the absence of drug selection, vector and transgene-free hiPSCs can be isolated through subcloning without further genetic manipulation. EV is now regarded as one of the most cost-effective and successful tools for reprogramming of cells of different tissue origins from adult donors [[91]–[99]].

### Modified mRNAs

Warren et al. described a simple, non-integrating strategy for reprogramming cells based on the administration of a synthetic mRNA that is modified to overcome innate anti-viral responses [[38],[100],[101]]. They synthesized mRNAs incorporating modified ribonucleoside bases, encoding KSOM, and including a modified-RNA encoding LIN28 to form a five-factor cocktail (KMOSL) with the ability to reprogram four human fibroblasts. This system has an efficiency of over 2%, two orders of magnitude higher than those typically reported for virus-based derivations. Moreover, the resultant iPSC colonies emerge as early as 17 days, in contrast to virus-mediated iPSC derivations, which typically take four weeks. The efficiency of HNFs transfected with either KMOS-modified RNAs or transfected with the KMOS retroviruses is 1.4% and 0.04%, respectively.

### Minicircle DNA

Minicircle DNA is a novel compact vector free of bacterial DNA and capable of sustaining high-level expression within cells. Jia et al. [[39]] constructed a plasmid (P2PhiC31-LGNSO) containing a single cassette of OSNL plus a GFP reporter gene, each separated by self-cleaving peptide 2A sequences. This system yields an overall reprogramming efficiency of ~0.005% in hASCs with minicircle DNA [[102]]. This efficiency is higher than that of previous plasmid-based transfection reprogramming methods, although this may be in part due to differences in donor cell types (HNFs vs. hASCs) and the number of reprogramming factors used. They then transfected HNFs with a minicircle vector and attained 10-fold less efficiency.

Although its efficiency is very low, minicircle DNA has its advantages. Compared with other iPSC derivations that are free of foreign or chemical elements, minicircle DNA is a simple method from adult donor sources, requiring only a single vector without the need for subsequent drug selection, vector excision, or the inclusion of oncogenes such as SV40. Finally, minicircle DNA is already FDA approved, giving this novel method the potential for significant clinical translation.

### HACs

HACs possess several characteristics that are required for gene therapy, including stable episomal maintenance and the capacity for large gene inserts. HACs can also carry genomic loci with regulatory elements, thus allowing for the expression of transgenes in a genetic environment similar to a chromosome [[103]].

Hiratsuka et al. devised a reprogramming cassette with four defined reprogramming factors and introduced multiple copies of the cassette into the cloning site of a HAC vector [[40]]. This HAC vector encodes herpes simplex virus thymidine kinase (HSV-TK) such that any produced iPSCs and/or their HAC-carrying differentiated derivatives can be killed by ganciclovir (GCV). This provides a safeguard system lest any unexpected events (e.g., tumor formation) occur. In addition, their HAC vectors encode EGFP. Because HACs are lost spontaneously at a low frequency, HAC-free cells can be isolated from reprogrammed iPS populations by identifying EGFP-negative cells. The overall efficiency of reprogramming achieved with the HAC strategy was approximately 0.001%.

### Protein delivery systems

Zhou et al. introduced a method using recombinant proteins to induce iPSC formation [[36]]. To generate recombinant proteins that can penetrate the plasma membrane of somatic cells, they designed and fused a poly-arginine (i.e., 11R) protein transduction domain to the C-terminus of OSKM. Four repeated protein transductions of reprogramming proteins, with the help of VPA, could reprogram the OG2/Oct4-GFP reporter MEF cells into iPSCs with an efficiency of 0.006%; for comparison, this efficiency is only 0.001% with proteins OSK.

This protein transduction method represents a significant advancement in generating iPSCs and has several major advantages over previous methods. First, it rules out any risk that the target cell genome will be modified by transgenes and consequently offers a method to generate safer iPSCs. Second, the protein transduction method provides a substantially simpler and faster approach than the genetic method, which requires time-consuming sequential selection of potentially integration-free iPSCs. Finally, given the robustness and wide availability of large-scale recombinant protein production, this completely chemically defined reprogramming regimen could potentially enable broader and more economical application of reprogramming methodologies.

Kim et al. reported the generation of stable iPSCs from human fibroblasts by directly delivering proteins OSKM fused with a cell-penetrating peptide (CPP) in HEK293 cell extracts [[37]]. When the HNFs are treated with cell extracts, efficient intracellular translocation of each recombinant protein was observed within eight hours. Repeated protein treatment cycles (with 16 hours of protein treatment followed by six days of incubation in ES media) could yield hiPSCs starting from the 6th cycle. Overall, the establishment of these hiPS-like colonies took approximately eight weeks, approximately doubling the time required for viral transduction. At present, the efficiency of iPSC generation is significantly lower using this protein-based protocol (approximately 0.001%). In particular, the whole protein extracts used in the present study limited the concentrations of factors delivered into the target cells, suggesting that iPSCs may be more efficiently generated using purified reprogramming proteins.

Compared to the work of Zhou et al., who reported that mouse iPSCs were not generated when only recombinant proteins were used, the system of Kim et al. generated hiPSCs with the direct delivery of reprogramming proteins in the absence of any chemical treatment. One possible explanation for these differences is that Kim et al. used reprogramming proteins expressed in mammalian cells, whereas Zhou et al. used refolded proteins after their expression in E. coli.

In general, protein-based iPSC generation requires either chemical treatment (e.g., VPA) or greater than four rounds of treatment. Furthermore, protein-based methods necessitate expertise in protein chemistry and handling, which are skills that many laboratories do not have. For these reasons, recombinant proteins are challenging to generate and purify in the quantities required. Lastly, low iPSC derivation efficiencies have been observed, presumably due to weak or non-constant expression of reprogramming factors.

### Small-molecule compounds

In their search for chemicals that could replace the transgenes needed to reprogram iPSCs, Hou et al. showed that pluripotent stem cells can be generated from mouse somatic cells at a frequency of up to 0.2% using a combination of just seven small-molecule compounds [[41]]. They confirmed for the first time that by using small molecules, exogenous “master genes” are dispensable for cell fate reprogramming. Small molecules have many advantages because they can be cell permeable, non-immunogenic, more cost-effective, and more easily synthesized, preserved, and standardized. Moreover, their effects on inhibiting and activating the function of specific proteins are often reversible and can be finely tuned by varying their concentrations. This chemical reprogramming strategy has powerful potential for use in generating functional desirable cell types for future clinical applications. However, current reprogramming methods that do without transcriptional factors have relatively low and inconsistent efficiency. The timing and dosage of specific small molecules need to be optimized. Moreover, the generation of iPSCs from human somatic cells using this strategy has not yet been reported.

### Perspectives

Successfully reprogramming somatic cells to a pluripotent state represents a significant advancement in stem cell research. To determine the range of tissue types amenable to reprogramming and their particular characteristics, to reduce tumorigenesis risk of transgene integration, and to increase the efficiency of induction, many efforts have been put on the selection of ideal donor cells and optimization of iPSCs approaches. The huge potential implications of disease-specific or patient-specific iPSCs have impelled scientists to solve problems hindering the applications of iPSCs in clinical medicine. The development of iPS technology towards clinical application involves a thorough investigation of the most favorable tissue origin for iPSCs generation. Although fibroblasts are the first cell type from which iPSCs are developed and are still being widely used, they are not the ideal source. The procedure requires biopsy and is contraindicated in life-threatening skin diseases. To date, cells from all three embryonic germ layers have been analyzed by comparing with fibroblasts, and advantages of some tissue origins over fibroblasts were identified. PBSs and HUCs are the most promising sources for iPSCs, but each of them has its own limitations. The reprogramming efficiency of PBSs is extremely low (0.0008 to 0.001%), and the mature T cells derived iPSCs harbor germ line IgH and TCR alleles; moreover, in some cases of infection and blood diseases, giving blood is not exempt of concerns. HUCs seem to be more promising than PBSs, they are non-invasive and can be reprogrammed with a high efficiency even with an integration-free reprogramming method (0.2%), However, other reports indicate a high degree of variability of excreted cell number in urine among individuals, and the proliferation was achieved in only approximately 1/3 of samples [[55]]. Moreover, culturing HUCs usually requires long periods of time (at least two weeks) [[53]]. Furthermore, iPSCs derived from this source are not fully analyzed so far regarding genomic stability and differentiation potential. Thus, further efforts are still needed to identify the ideal tissue for iPS generation that would be effective, safe, and convenient. A second major breakthrough is the elimination of the integration of the viral transgenes into the somatic genome. Current methods for iPSC induction can be divided into four categories based on their delivery system: viruses, DNA and RNA (plasmid, episomal plasmid, and transposon), cell-penetrating peptides, and chemicals. Another classification is based on genome-integration: integration, integration but excisable, and integration-free methods. Non-viral delivery integration-free methods, such as plasmid [[34]], EV [[35]], proteins [[36],[37]], and chemicals [[41]], have been successfully employed to produce integration-free iPSCs, albeit at a lower efficiency than retroviral and lentiviral methods. The reasons for the low efficiency of iPSC induction are unclear, but it may be that the precise balance between transgenes and/or the expression level of each transgene is important for reprogramming [[18]]. Generally, EV is now regarded to be one of the most cost effective, non-viral, integration-free vectors at present. It should be emphasized that the small molecule strategy is much more promising because of its outstanding advantages. Small molecule compounds can be cell permeable, non-immunogenic, and more easily synthesized, preserved, and standardized. Moreover, their effects on the function of specific proteins are often reversible and cell fate can be manipulated in a controlled microenvironment. This strategy could be more cost-effective if the efficiency of could be improved.

It is far too early to know for sure whether hiPSCs are safe for use in medical applications. Although hiPSC lines generated by integration-free methods are not found to have integrated transgenes by Southern blot analyses and PCR analyses, small pieces of vectors and transgenes could be inserted into the genome but not observed, given the detection limits of ordinary methods. Thus, because it is important to validate the quality of iPSCs, extensive genetic screening should become a standard procedure to ensure the safety of hiPSCs prior to their clinical use. Another pitfall that was discovered recently is potential immunogenicity against transplanted iPSCs. Immune tolerance had been considered an advantage of autologous cell transplantation derived from patient iPSCs. However, it is found that some certain but not all tissues derived from iPSC-derived cells can be immunogenic in syngenic hosts by using a teratoma transplantation model. The reasons for this phenomenon are very complicated, and both genetic and epigenetic defects in iPSCs could contribute either directly or indirectly [[104]]. In vivo immunogenicity testing should be used as a screening platform to improve the reprogramming technology [[105]].

## Conclusions and future directions

**Figure 1:**
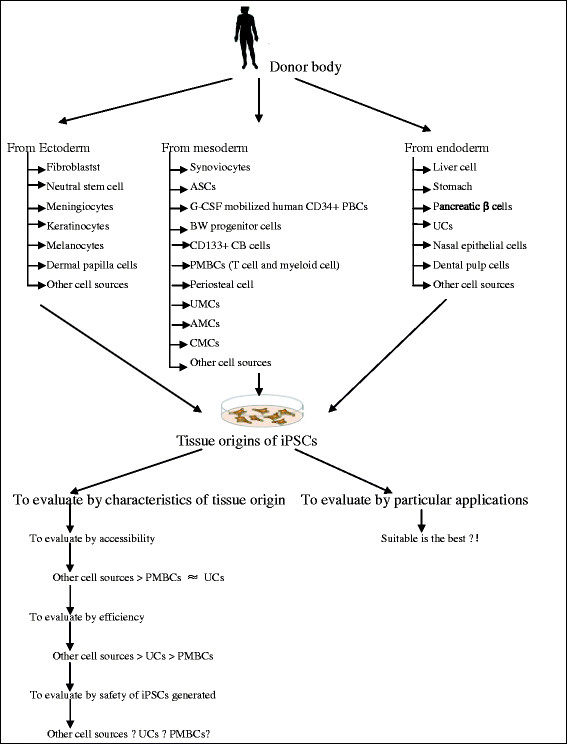
**A proposed strategy for the tissue origins of iPSCs.** Cells from three embryonic germ layers have been assessed through enormous research efforts, and the advantages that some tissue origins have over fibroblast origins concerning efficiency and accessibility have been elucidated. PBSs and HUCs are the most promising sources for iPSCs, but each of them has its own limitations. Further efforts are still needed to identify the ideal tissue for iPS generation that would be effective, safe, and convenient.

**Figure 2:**
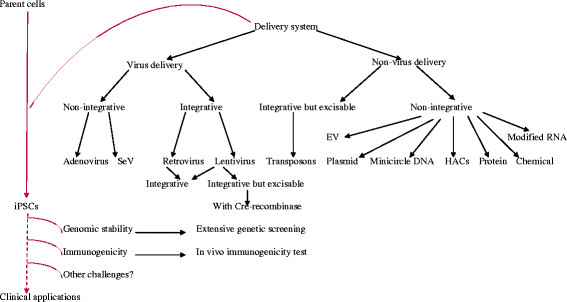
**Schematic representation of delivery systems used for iPS induction: viral, non-viral; integrative, integration but excisable, integration-free approaches and the challenges encountered.** Non-viral delivery integration-free methods have been successfully employed to produce integration-free iPSCs. Insertion of small pieces of vectors and transgenes into the genome of “integration-free” hiPSC lines and potential immunogenicity against transplanted iPSCs as well as other challengers prevent iPSCs from clinical applications.

Generally speaking, researches to develop new methods for iPS cell generation for clinical applications are an ongoing process. The range of tissue origins seems to have been extensively investigated, but we still have to find an ideal one for real practical application; rather, it is best to find one that most suit for a given application. For instance, PBSs which possess strong blood-forming potential are attractive for hematologists whereas DP cells maybe a better cell source for dermatologists to grow hairs. More efforts should be put on the improvement of the culture conditions and technology (see Figure 1). As for the approaches used to generate iPSCs, although they have been improved from viral integration to integration-free, there are still many challenges down the road to achieve their clinical application in humans (see Figure 2). It counts on further improvement of current reprogramming technology to minimize the genetic and epigenetic difference between iPSCs and donors. In addition, a more comprehensive understanding of the reprogramming process will be crucial for the development of future approaches for clinical applications of iPSCs.

## Abbreviations

iPS cells or iPSCs: Induced pluripotent stem cells

ESCs: Embryonic stem cells

MEFs: Mouse embryonic fibroblasts

TTFs: Tail-tip fibroblasts

O: Oct 3/4

S: Sox2

K: Klf4: M, c-Myc

hiPSCs: Human iPSCs

HDFs: Human dermal fibroblasts

HFLS: Human fibroblast-like synoviocytes

N: Nanog

L: Lin28

SeV: Sendai virus

miRNAs: microRNAs

oriP/EBNA1: Epstein-Barr nuclear antigen-1

EV: Episomal vector

IRES2: Internal ribosome entry site 2

SV40LT: SV40 large T gene

VPA: Valproic acid

Vc: Vitamin C

siRNA: Small-interfering RNA

hASCs: Human adipose stem cells

GFP: Green fluorescent protein

mRNA: Messenger RNA

CNVs: Copy number variations

PB: piggyBac

DP cells: Dermal papilla cells

BM: Bone marrow

CB: Umbilical cord blood

G-CSF: Granulocyte colony stimulating factor

PBCs: Peripheral blood cells

TCR: T cell receptor

HUCs: Human urine-derived cells

NSCs: Neural stem cells

dox: Doxycycline

CMCs: Chorionic mesenchymal cells

UMCs: Mesenchymal-like cells from the umbilical cord matrix, AMCs, the amniotic membrane mesenchyme

GSK-3: Glycogen synthase kinase 3

SFK: pan-Src family kinase

PCR: Polymerase chain reaction

bp: Base pairs

LTR: Long terminal repeat

HACs: Human artificial chromosome vectors

HNFs: Human newborn fibroblasts

HSV-TK: Herpes simplex virus thymidine kinase

CPP: Cell-penetrating peptide

## Competing interests

The authors confirm that this article content has no conflicts of interest.

## Authors’ contributions

JL and WS carried out the primary literature search and drafted the manuscript. GP and JZ provided material input and helped in revising the manuscript. WS and GP conceived the study and provided field expertise. All authors read and approved the final manuscript.
